# Supplementary material for: The allocation of carbon resources in marine capture fisheries
Source: PLoS One. 2024 Mar 15;19(3):e0293120. doi: 10.1371/journal.pone.0293120 (PMC10942046; doi:10.1371/journal.pone.0293120)
Supplement: Attachment — Submitted filename: Response to ReviewersR2.docx [file pone.0293120.s002.docx]

Reviewer1

Thank you for reviewing the manuscript.

Reviewer2

Thank you for reviewing the manuscript.

Reviewer3

Thank you very much for your comments. The paper has adjusted the tables2,tables4 and Figure 4.
